# Supplementary material for: Data-driven prediction of colonization outcomes for complex microbial communities
Source: Nat Commun. 2024 Mar 16;15:2406. doi: 10.1038/s41467-024-46766-y (PMC10944475; doi:10.1038/s41467-024-46766-y)
Supplement: Supplementary file 1 — Supplementary Information [file 41467_2024_46766_MOESM1_ESM.pdf]

Supplementary Information

Data-driven prediction of colonization outcomes for complex microbial communities

Lu Wu, Xu-Wen Wang *et al.*

Corresponding authors: Yang-Yu Liu, yyl@channing.harvard.edu; *Lei Dai*, lei.dai@siat.ac.cn

The PDF file includes:

Supplementary Methods
Supplementary Note 1
Supplementary Figs. S1 to S24
Supplementary Table S1
Supplementary References

Supplementary Methods

In silico simulations of colonization outcomes

We generated synthetic data of colonization outcomes using the generalized Lotka–Volterra (GLV) model[1]:

$$\frac{dx_i(t)}{dt} = x_i(t)\left[r_i + \sum_{j=1}^{N} a_{ij}x_j(t)\right], i = 1, \cdots, N. \quad (1)$$

Here $x_i(t)$ represents the absolute abundance of the *i*-th species at time $t \geq 0$. The pair-wise microbial interaction is presented by the matrix $A = (a_{ij}) \in \mathbb{R}^{N\times N}$, with $a_{ij} > 0$ (< 0, or $= 0$) means that species-j promotes (inhibits or does not affect) the growth of species-i, respectively. The diagonal elements of A are set to be $a_{ii} = -1$to ensure the stability of the system. The ecological network $G(A)$ is constructed using an Erdős-Rényi random graph model[2] with N nodes (i.e., species) and connectivity C (i.e., the probability connecting two species). To generate the interaction matrix A of ecological network, for each link $(j \to i) \in G(A)$ with $j \neq i$, we draw a_{ij} from the normal distribution $\mathbb{N}(0, \sigma)$. All other entries of A are set to be zero. The intrinsic growth rate vector $r = [r_i] \in \mathbb{R}^N$ is drawn from a uniform distribution $\mathcal{U}(0,1)$. Each local community includes N_s species randomly drawn from the $(N-1)$ species (excluding the exogenous species) and N_s=30 in all simulations.

To examine the performance of colonization outcome prediction in communities with varying levels of network complexity, we tuned the network connectivity C from the set [0.3, 0.4, 0.5]. In addition, to evaluate the sample size required for accurate prediction, we systematically tuned the size of training samples S_{train}/N from 0.5 to 10. An independently generated set of 100 samples were used as test data to evaluate the models. To generate the training samples for classification, we selected 1,100 local communities where the post-invasion steady-state absolute abundance of the exogenous species is above 0.05 (i.e., the threshold used to determine successful colonization) in half of the local communities, and below 0.05 in the other half. To generate the training samples for regression, we selected 1,100 local communities in which the post-invasion steady-state abundance of the exogenous species follows the log-normal distribution (mean=-3, standard deviation=0.5).

Colonization outcome prediction by machine learning models

We developed a deep learning model for Colonization Outcome Prediction using the Neural Ordinary Differential Equations (COP-NODE)[3]. The architecture of COP-NODE consists of two fully collected layers, and each fully connected layer (with dimension N) is followed by a normalization layer and a ReLU activation layer. The final layer is Sigmoid activation. The Adam optimizer was used for the optimization with a learning rate 0.01 for both classification and

regression. The loss function is CrossEntropy for classification and SmoothL1Loss for regression[4]. We randomly selected 20% of training samples as the validation set to select the best model and hyperparameters. For classification, we tuned the batch size from the set [16, 32, 64] and the hyperparameter β (the threshold to change between L1 and L2 regularization) from the set [0.001, 0.01, 0.1, 0.2, 0.4, 0.6, 0.8, 1]. Other machine learning models used in this study, including Logistic Regression, Elastic Net, Random Forest classifier, and regressor, were implemented using the Python package scikit-sklearn[5]. We used randomized search on hyperparameters and 3-fold cross-validation to optimize the AUROC for classification and R^2 for regression. The regression models were trained to predict the log-transformed abundance of the exogenous species.

Colonization impact of resident species onto the invading species

To compute the colonization impact, e.g., the impact of resident species onto the colonization outcome of the invading species, we first trained the prediction models using all the samples. Then, for resident species i in a permissive local community α, we performed a thought experiment by introducing a perturbation in the abundance of resident species i, and used the trained machine learning model to predict the new steady state abundance of invading species $\tilde{x}_i^\alpha$ after the perturbation. The perturbation is performed by increasing the abundance of a certain resident species by 0.007, which represents ~5% of the total biomass. The colonization impact (CI) of resident species i onto the invading species in local community α is defined as:

$$\mathrm{CI}_i^\alpha = \frac{\tilde{x}_i^\alpha - x_i^\alpha}{\tilde{x}_i^\alpha + x_i^\alpha}$$

where x_i^α is the steady state abundance of invading species in community α before perturbing the abundance of species i. A negative colonization impact ($\mathrm{CI}_i^\alpha < 0$) indicates that species i inhibits the colonization of the invading species in community α. For classification models, x_i^α and $\tilde{x}_i^\alpha$ represents the colonization probability before and after perturbing the abundance of species i, respectively.

Validation of the inhibitory effect of *E. faecalis* on *E. faecium* colonization

Pairwise co-culture experiments

Soft Agar Overlay Assays were conducted using BHI agar plate. *E. faecium* DA797 was cultured to an OD_{600} of 0.6 and 100ul of the inoculum was pipetted into 10mL prewarmed (42°C) BHI containing 0.75% (w/v) agar. The mixture was briefly mixed and then transferred onto a plate already laid with 10mL BHI 1.5% agar and four Oxford cups, to embed *E. faecium* into soft agar. The mixture was spread evenly on the surface of the plate. Next, 100-µl volumes of *E. faecium, E. faecalis* DA894, *E. faecalis* DA462 (OD_{600}=0.6) were added individually into the Oxford cups. The plates were incubated anaerobically at 37 °C for 24h before observation. The experiment was performed three times with two technical replicates for each strain.

Liquid co-culture experiments were performed in BHI at 37°C static, under anaerobic conditions. *E. faecium* and *E. faecalis* were cultured separately in BHI at 37°C for 24h without shaking, then diluted in BHI to an OD_{600} of 0.005 and then inoculated at 1:1 ratio into 1 mL of BHI broth and grown for 24h without shaking. Mono- and co-culture outputs were centrifuged to remove the supernatant, and the pellets were subsequently DNA extracted and *E. faecium* specific qPCR primer was used to detect the abundance of *E. faecium*.

<u>Community experiments</u>

Frozen stocks of *E. faecium* DA797, *E. faecalis* DA462 and DA894 and *C. symbiosum* DA229, were grown anaerobically at 37 °C in BHI until they reached the stationary phase. Eight baseline communities' stocks were revived into 980μL MiPro medium with three replicates in deep well plates. After 24h's incubation at 37 °C, the community biomass was measured by OD_{600}. Saturated cultures were then diluted 5μL into 1mL of fresh MiPro in a new 96-well plate before the invasion experiments. Three different experimental schemes were used: 1) Add *E. faecalis* (or *C. symbiosum*) into the baseline community, followed by *E. faecium* on the next day; 2) Add *E. faecalis* and *E. faecium* on the same day; 3) Add *E. faecium* into the baseline community, followed by *E. faecalis* on the next day. The inoculum was incubated at 37 °C and serially diluted every 24 h of 7 passages until the community reached a steady state. Saturated cultures were centrifuged to remove the supernatant, and the pellets were stored at -80°C with a plastic seal until DNA extraction. *E. faecium* abundance was assessed by both metagenomic sequencing and qPCR.

<u>qPCR assays for absolute quantification</u>

qPCR reactions were used to validate the impact of *E. faecalis* on the colonization outcome of *E. faecium*. qPCR reactions (0.5 μl DNA, 0.2 μM each primer, Hieff® qPCR SYBR Green Master Mix (Yeasen) were performed on a Bio-Rad CFX384 Touch Real-Time PCR Detection System, using primers specific for *E. faecium* under the following reaction conditions: 95 °C for 5min followed by 40 cycles of 95 °C for 10s , 60°C for 20 s and 72°C 20 s. *E. faecium*-specific primer sequences were: Ala-F:ATCCCTCTGGGCACGCAC, Ala-R:ACATACACGCCCAATCGTTTC, as described previously [6]. Standard curves using genomic DNA of *E. faecium* were used for absolute quantification of *E. faecium* copy numbers.

Estimating the absolute abundance of stool-derived microbial communities through optical density (OD_{600}) Measurements. To verify whether OD_{600} is an effective measure for estimating microbial total biomass, we conducted experiments to assess the correlation between OD_{600} readings and cell density in stool-derived communities (see **Fig.S24**). Six different stool-derived microbial communities were thawed and revived by adding 20 μL of the stocks to 980 μL of BHI medium in deep well plates. After incubation for 24 hours at 37°C, the saturated cultures were diluted into 1 mL of BHI in a new plate at five different dilution ratios (1:1.5, 1:2, 1:4, 1:8 and

1:16). Subsequently, the OD_{600} of these diluted communities was measured using an Epoch 2 plate reader (BioTek). Parallelly, we assessed the particle density of these communities using CytoFLEX s cytometry (Beckman Coulter). Our findings reveal a strong correlation between OD_{600} values and cell densities determined via flow cytometry, supporting the use of OD_{600} as a reliable method for estimating the absolute abundance of stool-derived microbial communities in our study.

E. faecium and *E. faecalis* abundance analysis in human cohorts

The following datasets were used for the metagenomic analysis of the species of interest in four large and diverse human cohorts: Israel[7], Lifelines-DEEP[8], PERDICT-1[9], TwinsUK[10] and SIAT cohort. Sequencing data were obtained using the accession numbers provided in the associated references and processed by SHOGUN pipeline as previously described. *E. faecium* and *E. faecalis* with relative abundance below 0.0001 is set to 10^{-4} for visualization.

Supplementary Note 1

Analytical derivation on the steady state abundance of exogenous species in GLV model

For a local community α of s resident species governed by GLV dynamics, we denote the post-invasion steady state abundance of the exogenous species as $x_{s+1}^{(1)}$. After invasion, the community arrives at a new steady state, i.e., $\frac{dx_{s+1}(t)}{dt} = 0$. Thus, according to Eq.1, $x_{s+1}^{(1)}$ can be expressed as:

$$x_{s+1}^{(1)} = r_{s+1} + \boldsymbol{c}\mathbf{x}_{1:S}^{(1)}. \qquad (2)$$

Here, the s-dimensional vector $\boldsymbol{c}$ represents the interaction strength of the resident species onto the exogenous species, $\mathbf{x}_{1:S}^{(1)}$ represents the post-invasion steady state abundance of the resident species.

Based on derivations in our previous study[11], the shift in the steady state abundance of resident species (i.e. the difference between $\mathbf{x}_{1:S}^{(1)}$ and the pre-invasion steady state $\mathbf{x}_{1:S}^{(0)}$) satisfies the following relation:

$$\mathbf{x}_{1:S}^{(1)} - \mathbf{x}_{1:S}^{(0)} = -\boldsymbol{A}^{-1}\boldsymbol{b}x_{s+1}^{(1)}. \qquad (3)$$

Here, the s-dimensional state vector $\mathbf{x}_{1:S}^{(0)} = [\mathbf{x}_1^{(0)}, \mathbf{x}_2^{(0)}, \cdots \mathbf{x}_S^{(0)}]^{\mathrm{T}}$ represents the pre-invasion steady state of the local community, the s-dimensional vector $\boldsymbol{b}$ represents the interaction strength of the exogenous species onto the resident species. The interactions among resident species are encoded in matrix $\boldsymbol{A}$.

By combining Eq.2 and Eq.3, we can derive a surprisingly simple linear relation between the post-invasion abundance of the exogenous species $x_{s+1}^{(1)}$ and the pre-invasion abundance of resident species $\mathbf{x}_{1:S}^{(0)}$:

$$x_{s+1}^{(1)} = \frac{r_1 + \boldsymbol{c}^{\mathrm{T}}\mathbf{x}_{1:S}^{(0)}}{1 + \boldsymbol{c}^{\mathrm{T}}\boldsymbol{A}^{-1}\boldsymbol{b}}. \qquad (4)$$

This analytically derived relation can fully explain the simulated colonization outcomes in the GLV model (**Fig.S1**, Spearman correlation $\rho = 1, p < 0.001$). We note that results similar to Eq. 4 have been utilized in the cavity method applied to the GLV model[12]. Although the linear relation in Eq. 4 doesn't hold for other dynamical models (e.g., non-linear interactions), it gives us important insights that learning the mapping for colonization outcome prediction is feasible by data-driven models and the number of parameters required for fitting the relation is on the order of *~O(N)*. This is consistent with our observations on the number of training samples required for accurate prediction of colonization outcomes (**Fig. 1**).

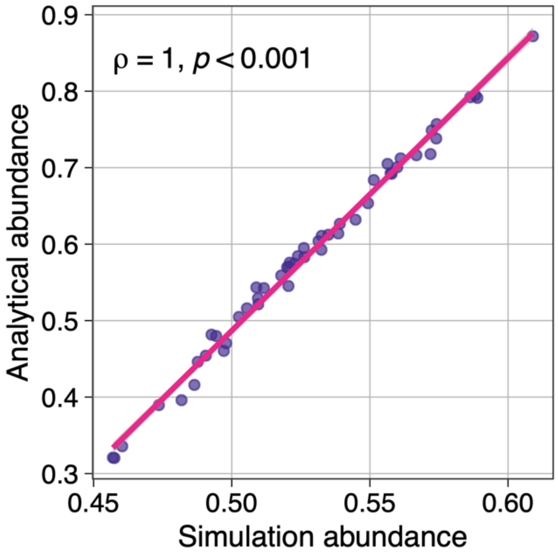

Fig. S1. The steady state abundance of an invading species in communities governed by GLV dynamics: comparison between analytical derivations and simulations. The analytically derived relation (Equation 4 in **Supplementary Text**) can fully explain the simulated colonization outcomes in the GLV model (Spearman correlation $\rho = 1, p < 0.001$). We generated 50 local communities, each consisting of 4 species randomly drawn from a meta-community of 7 species. Network connectivity $C = 1$ and interaction strength $\sigma = 0.2$. Species-8 was introduced as an invading species.

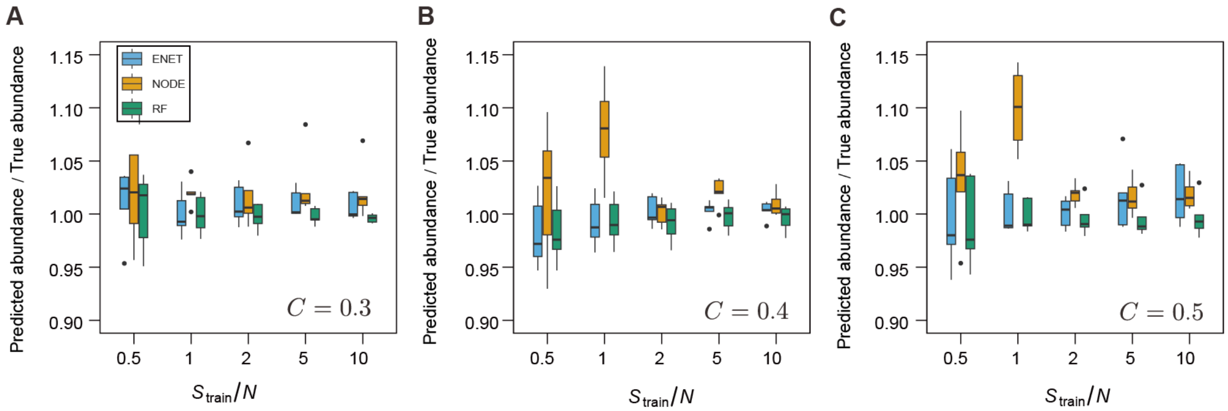

Fig.S2. The ratio between the predicted abundance and the true abundance. Evaluation of the data-driven approach in solving the regression task of COP. Pearson correlation between the true abundance and the abundance predicted by three machine learning models, including Elastic Net Linear Regression (ENET), COP-NODE regressor (NODE), and Random Forest regressor (RF) with network connectivity $C = 0.3$ (**A**) $C = 0.4$ (**B**) $C = 0.5$ (**C**).

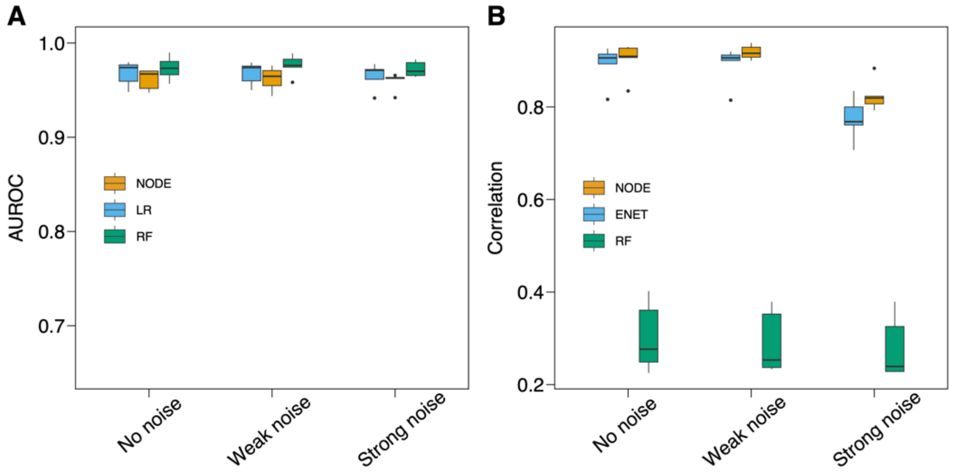

Fig.S3. Prediction of colonization outcomes in simulated data with noise. We first generated noise drawn from the normal distribution with mean 0 and standard deviation ε. Then, we added the noise into microbial composition: $\tilde{p} = \max(p(1 + \mathcal{N}(0, \varepsilon), 0)$, with ε representing the noise strength. We used three ε values corresponding to no noise ($\varepsilon = 0$), weak noise ($\varepsilon = 0.01$), and strong noise ($\varepsilon = 0.1$), respectively. **(A)** Evaluation of the data-driven approach in solving the classification task of COP. AUROC of three machine learning models, including Logistic Regression (LR), COP-Neural Ordinary Differential Equations classifier (NODE), and Random Forest classifier (RF). **(B)** Evaluation of the data-driven approach in solving the regression task of COP. Pearson correlation between the true abundance and the abundance predicted by three machine learning models, including Elastic Net Linear Regression (ENET), COP-NODE regressor (NODE), and Random Forest regressor (RF). The models were trained with 200 samples, and network connectivity $C = 0.3$.

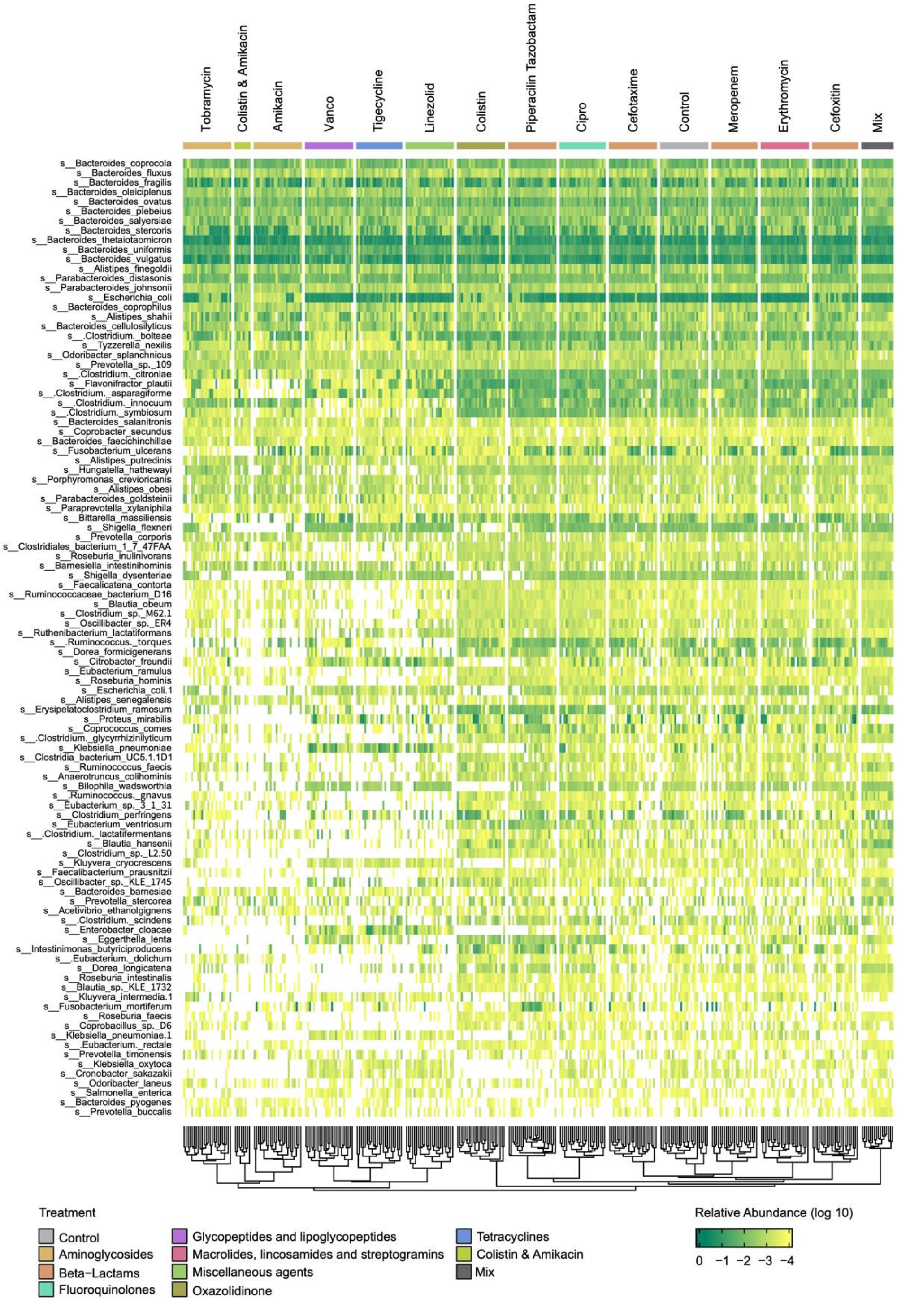

Fig. S4. The compositional profile of baseline communities at the species level. Each column corresponds to a baseline community derived from a human stool sample (24 donors) treated with antibiotics (12 antibiotics). Mix indicates the group of communities derived from mixing two different donors. Each row corresponds to a species, clustered by the similarity of relative abundance across baseline communities. Species with top 100 prevalence are displayed.

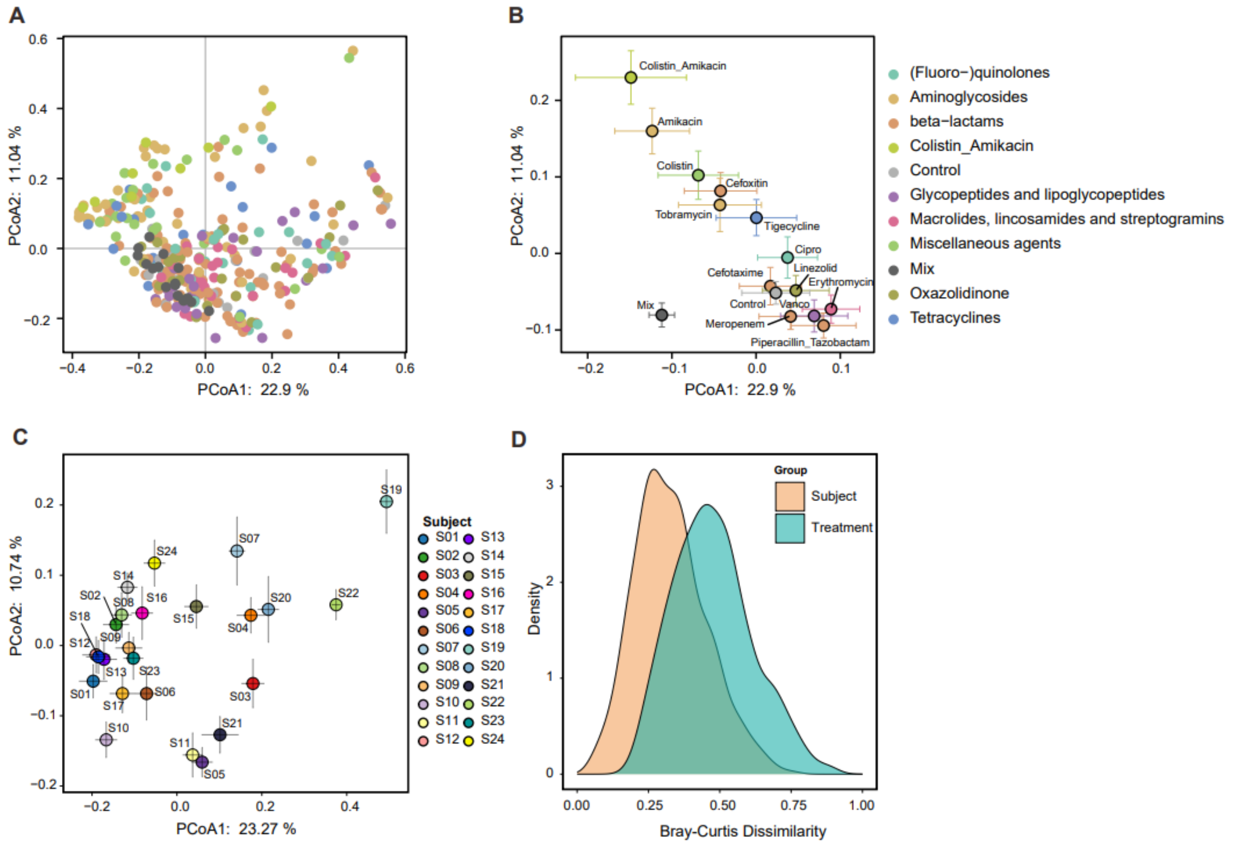

Fig. S5. Generation of diverse baseline communities by antibiotics treatments. (A) Principal-coordinate analysis (PCoA) based on the Bray-Curtis dissimilarity of the compositional profiles at the species level. The baseline communities are color-coded according to antibiotics treatments. **(B)** The colored dot for each antibiotics treatment represents the compositional profile averaged over 24 subjects. Error bars are SEMs. The antibiotics of different classes had distinct impacts on community structure. Tobramycin and amikacin, belonging to aminoglycosides, drastically changed the community structure. In contrast, meropenem, cefoxitin, and cefotaxime, belonging to beta-lactams, had relatively moderate impacts on the community structure. **(C)** The colored dot for each subjects represents the compositional profile averaged over antibiotics treatment. Error bars are SEMs. **(D)** The density plot illustrating Bray-Curtis dissimilarity in compositional profiles at the species level across various subjects and treatments.

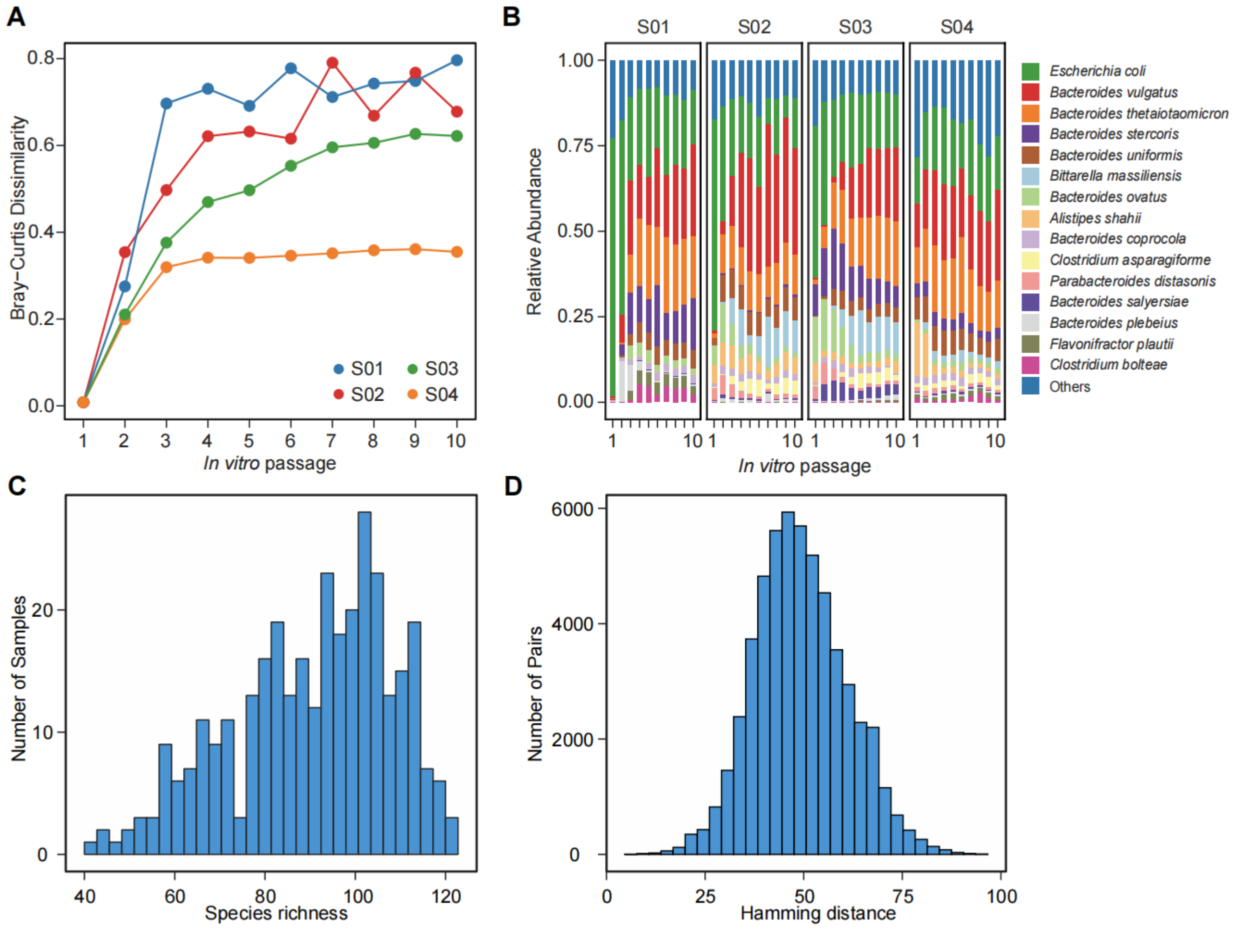

Fig. S6. Stabilization of human stool-derived *in vitro* communities and the statistics of steady-state baseline community composition. (A) The Bray-Curtis dissimilarity to the initial compositional profile during serial passaging. Colored lines indicate the trajectories of communities from different donors (S01-S04). **(B)** Time series of the compositional profiles. The human stool-derived *in vitro* communities reached steady states after ~5 rounds of serial passaging in the MiPro medium. **(C)** Species richness of steady-state baseline communities. **(D)** Hamming distance between the species presence/absence profiles of baseline communities.

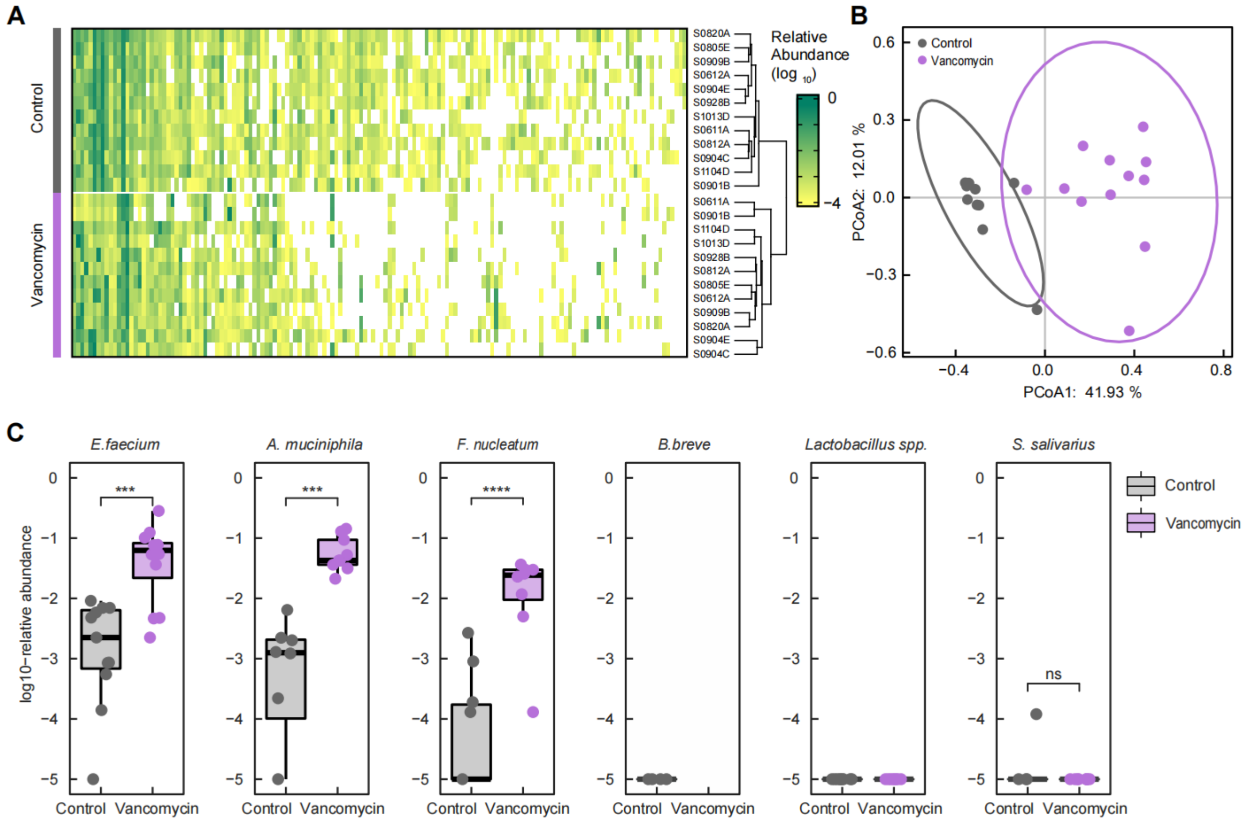

Fig. S7. Colonization outcomes of different exogenous microbial species in human stool-derived *in vitro* communities. (A) The compositional profile of baseline communities at the species level. Each row corresponds to a baseline community derived from a human stool sample (12 donors) treated with vancomycin (Vanco) or not (Control). Each column corresponds to a species, clustered by the similarity of relative abundance across baseline communities. Species with top 100 prevalence are displayed. **(B)** Vancomycin treatment altered the community structure at the species level (Adonis test, R^2=0.36， p<0.0001), as determined by PERMANOVA based on the Bray-Curtis dissimilarity. **(C)** Colonization outcomes of different exogenous species, including *E. faecium, A. muciniphila, F. nucleatum, S. salivarius, B. breve* and *Lactobacillus spp.* (*L. plantarum HNU082* and *L. paracasei HNU312*). The relative abundance of the invading species was determined by metagenomic sequencing of the final time point. We found that *E. faecium*, *A. muciniphila* and *F. nucleatum* could successfully colonize in some communities at varying levels of post-invasion abundance. In contrast, *S. salivarius, B. breve* and *Lactobacillus spp.* were unable to colonize in nearly all the baseline communities that we tested. Moreover, we found that vancomycin treatment significantly altered the colonization outcomes, rendering the communities more susceptible to invasion. ns, not significant, ***p < 0.001, ****p < 0.0001, Mann-Whitney U-tests. For visualization, the relative abundance was set to 10^{-5} if it was below the detection limit (i.e. failed invasion).

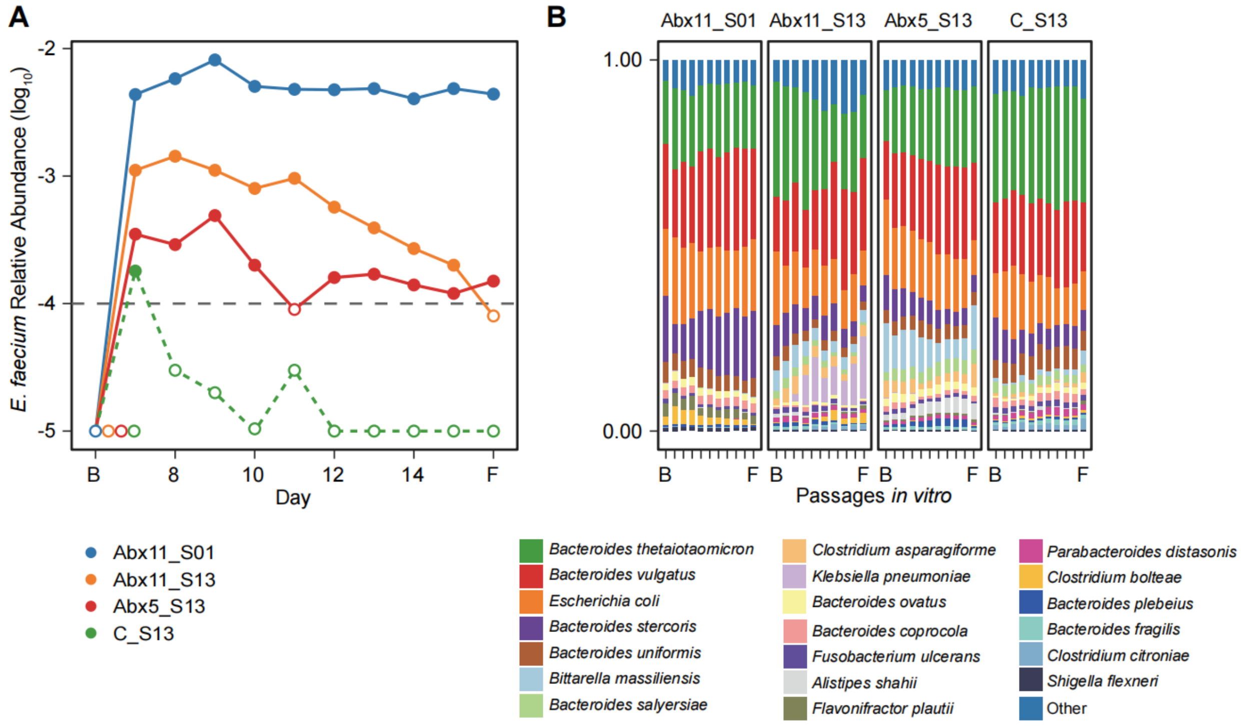

Fig. S8. Post-invasion time series of *E. faecium* abundance and community composition. (A) The colonization outcome of *E. faecium* in different communities was persistent during serial passaging. The dashed line indicates the detection limit of the relative abundance of *E. faecium* (**Fig.S23**). **(B)** The community composition was stable during serial passaging. B and F denote the baseline and the final time point.

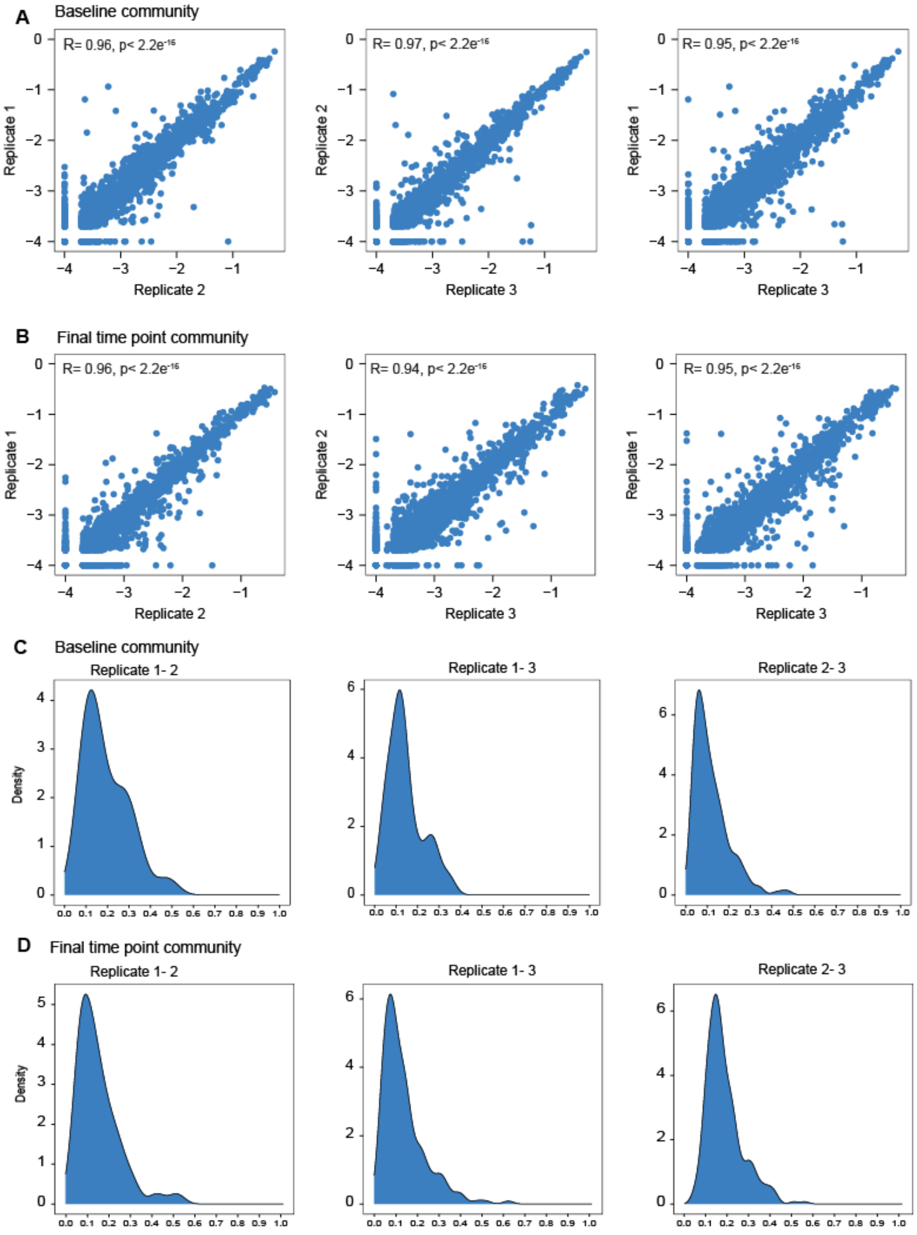

Fig. S9. The composition of *in vitro* communities before and post *E. faecium* invasion is highly reproducible across replicates. The species-level compositional profile of the baseline communities (**A**) and of the post-invasion communities (**B**) is highly reproducible among technical replicates (Pearson correlation). For visualization, the relative abundance was set to 10^{-4} if it was below the detection limit. n= 3 replicates. **(C-D)** Distribution of Bray-Curtis dissimilarity between replicates.

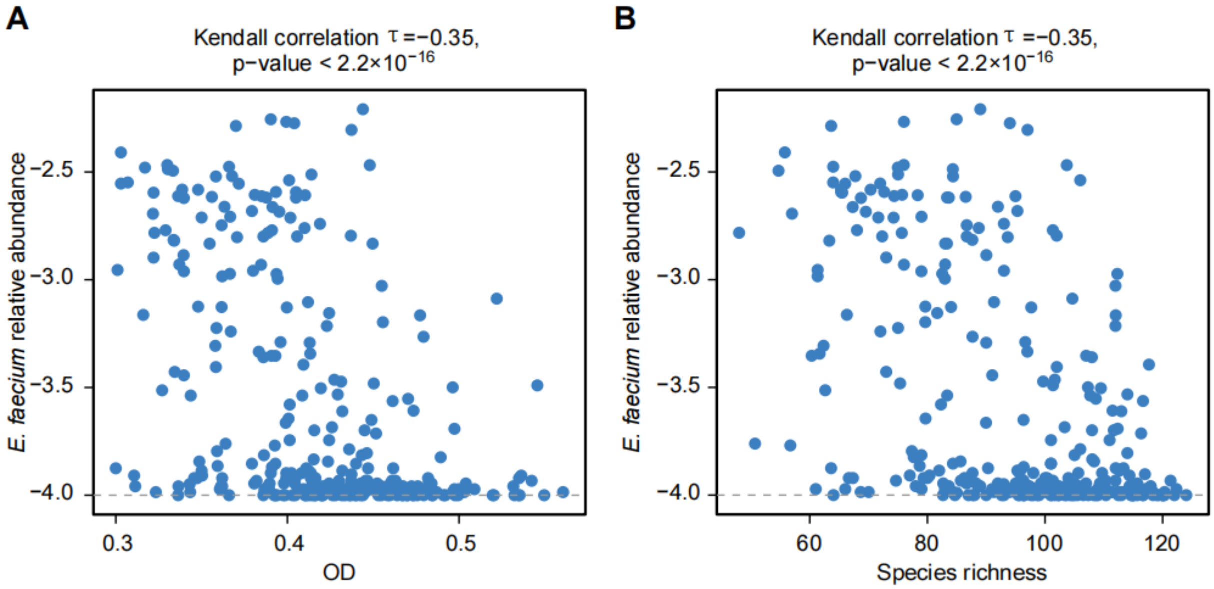

Fig. S10. The invasion resistance to *E. faecium* increases with community biomass and diversity. (A) The post-invasion steady state abundance of *E. faecium* is negatively correlated with the biomass of baseline communities (measured by OD_{600}). **(B)** The post-invasion steady state abundance of *E. faecium* is negatively correlated with the species richness of baseline communities.

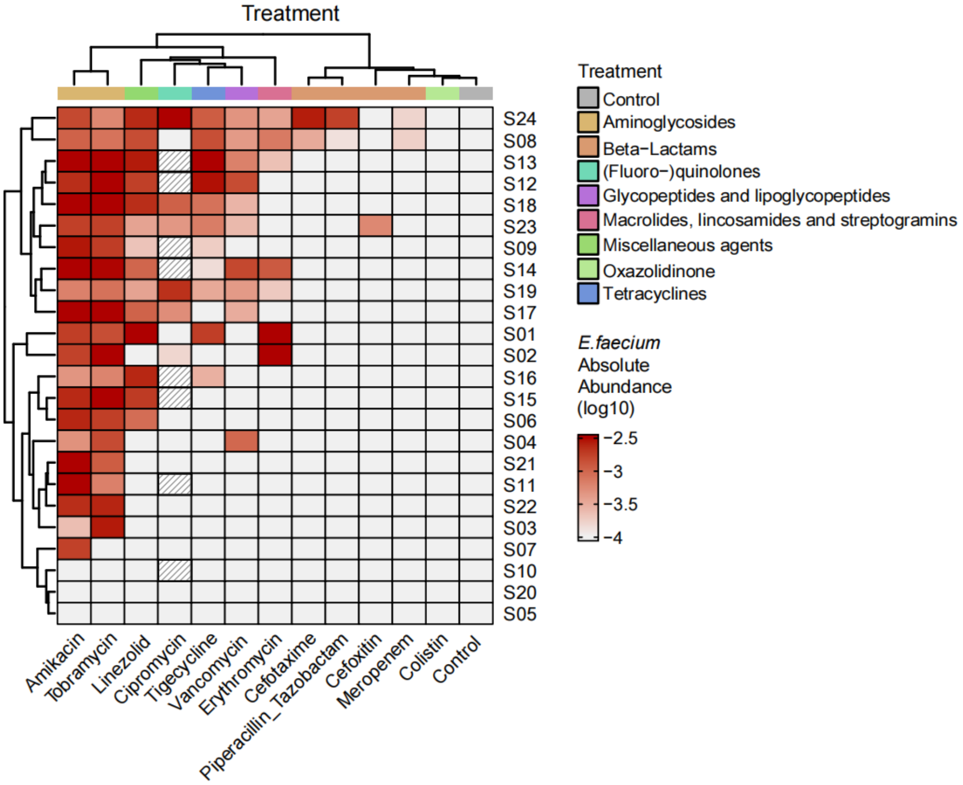

Fig. S11. Variations in the colonization outcomes of *E. faecium* across different donors and antibiotics treatments. For instance, post-invasion abundance of *E. faecium* in communities derived from donor S24 was higher than other donors; post-invasion abundance of *E. faecium* in communities treated with amikacin was higher than the control group and other treatment groups. Each row corresponds to a donor from which the communities were derived, each column corresponds to a treatment. The color gradient represents absolute abundance (OD_{600}×relative abundance) of *E. faecium* at the post-invasion steady state. Samples marked with slashes are not available.

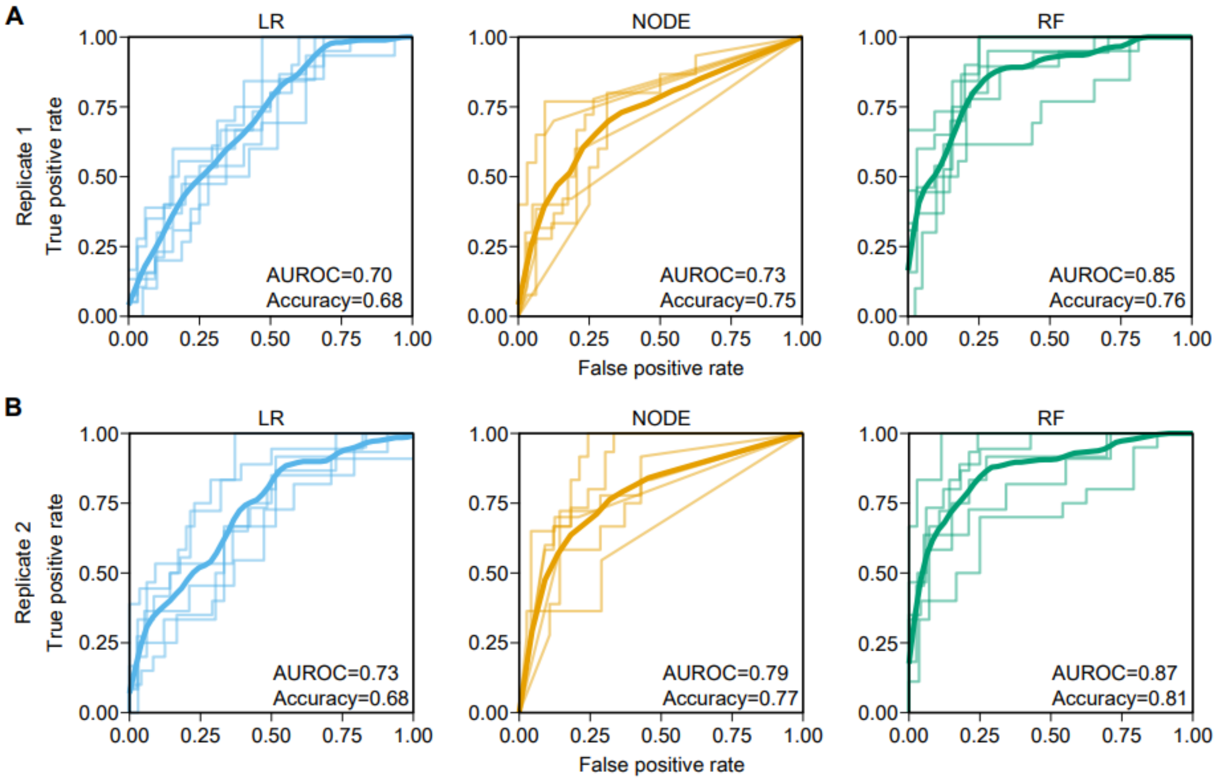

Fig. S12. The performance of colonization outcome prediction for *E. faecium* is consistent across replicates. ROC curve of machine learning models in binary classification (permissive vs. resistant) of the colonization outcomes of *E. faecium* in replicate 1 **(A)** and replicate 2 **(B)**. For each 6-fold cross validation (ROC curves shown in light color), we trained each model using the samples from 20 subjects and the samples from the remaining 4 subjects to evaluate the model. The mean ROC curve is shown in dark color. LR: Logistic Regression, NODE: COP-Neural Ordinary Differential Equations classifier, RF: Random Forest classifier.

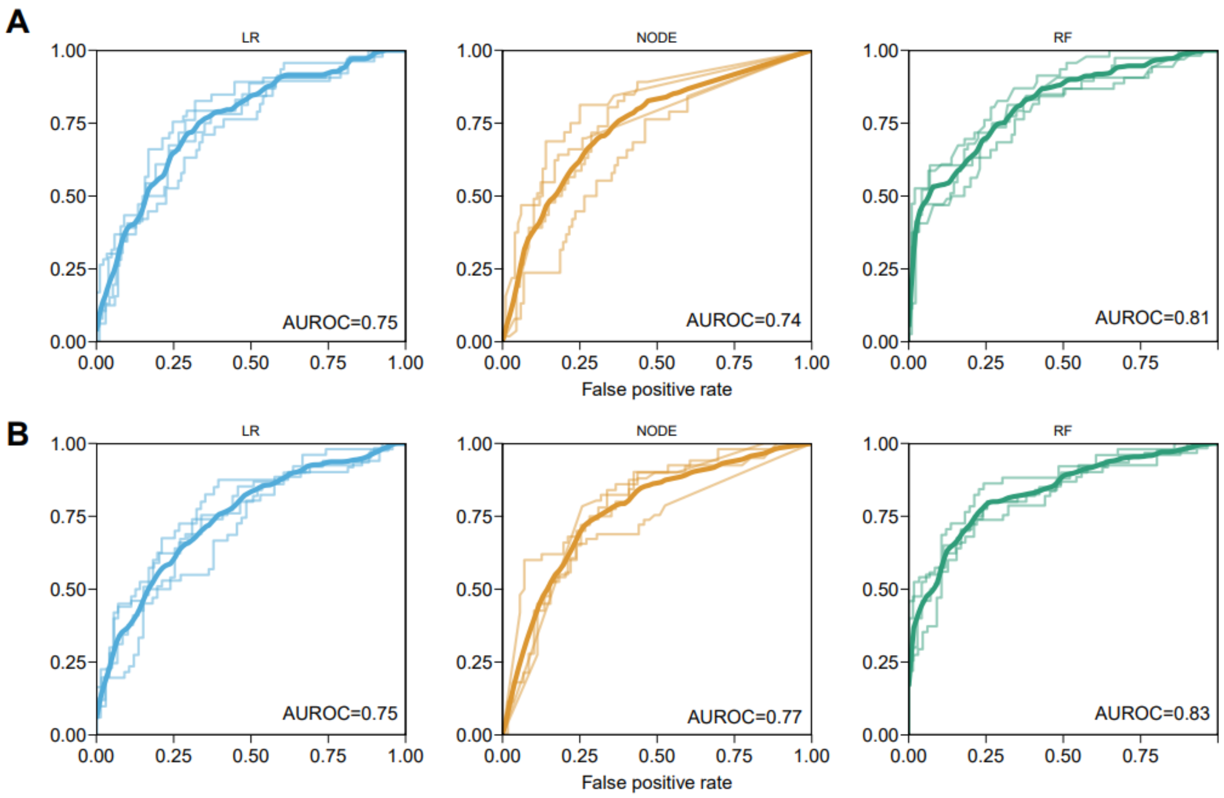

Fig. S13. The colonization outcome of *E. faecium* and *A. muciniphila* in human stool-derived *in vitro* microbial communities with a balanced training-test split. (A-B) ROC curve of machine learning models in binary classification (permissive vs. resistant) of the colonization outcomes of *E. faecium* (A) and *A. muciniphila* (B).

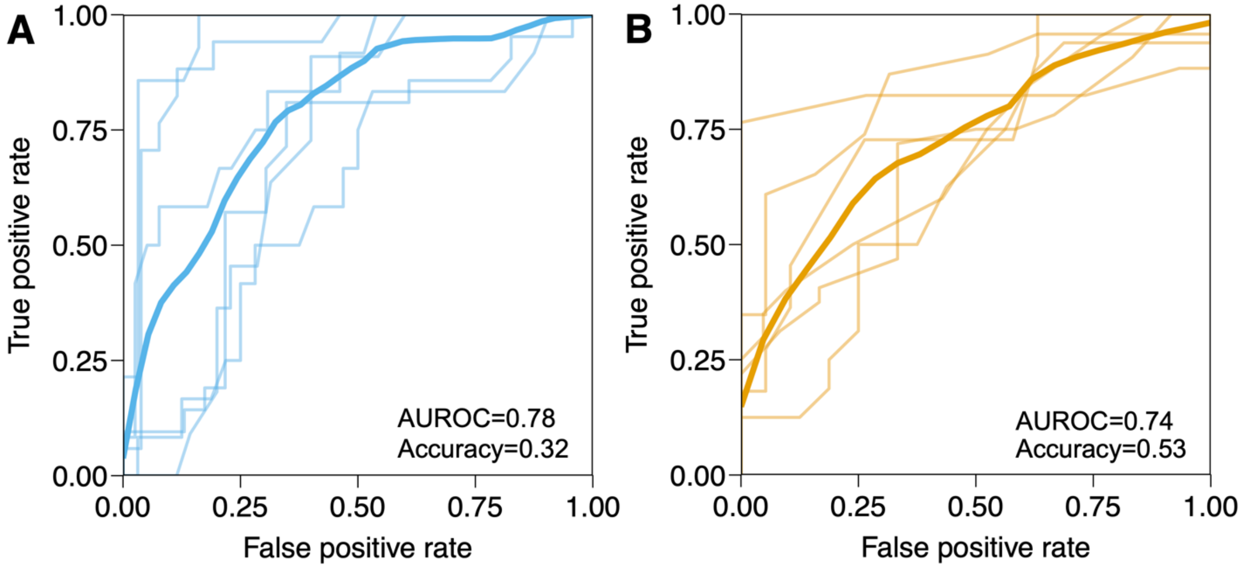

Fig.S14. The colonization outcome prediction of *E. faecium* and *A. muciniphila* in human stool-derived *in vitro* microbial communities using the community's relative species richness as the only predictor. Here, the relative species richness, denoted as r, of a microbial community is computed as the ratio between the number of species present in this community and that present in all baseline communities. For each of the 6-fold cross-validations (ROC curves shown in a light color), we simply used $(1 - r)$ as the colonization probability to compute its AUROC (or Accuracy). For colonization outcomes of *E. faecium*, the average AUROC (or Accuracy) based on the relative species richness is 0.78 (or 0.32), respectively. By contrast, the average AUROC (or Accuracy) of the Random Forest classifier based on the taxonomic profile is 0.86 (or 0.82), respectively (see **Fig. 3G**). For colonization outcomes of *A. muciniphila*, the average AUROC (or Accuracy) based on the relative species richness is 0.74 (or 0.53), respectively. By contrast, the average AUROC (or Accuracy) of the Random Forest classifier based on the taxonomic profile is 0.84 (or 0.78), respectively (see **Fig. 4G**).

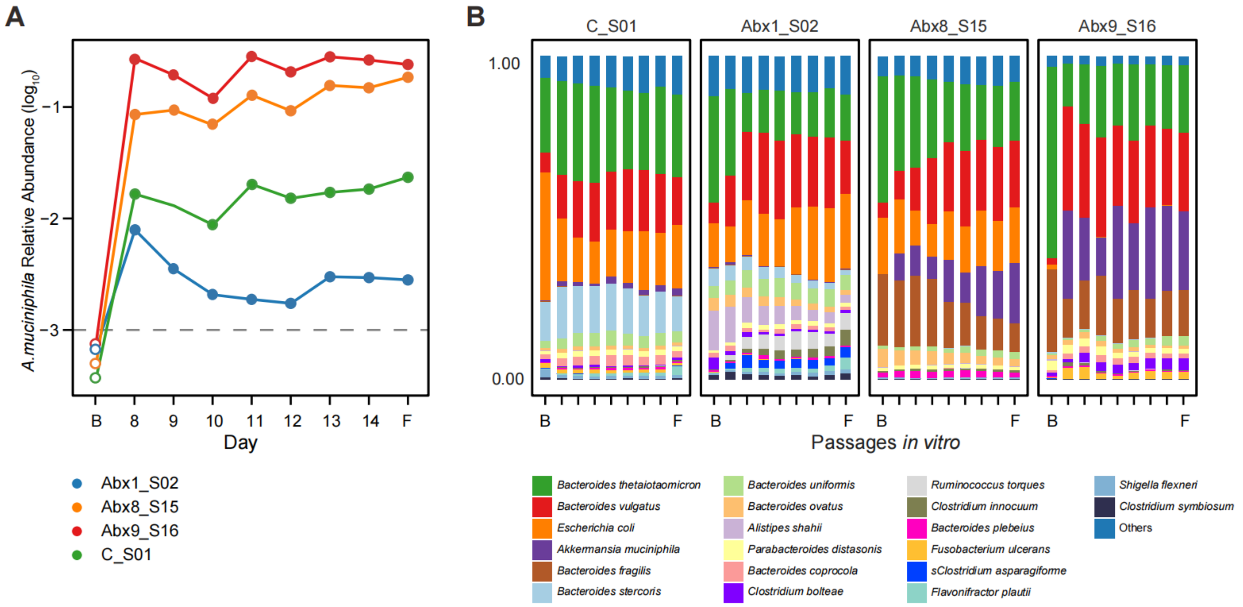

Fig. S15. Post-invasion time series of *A. muciniphila* abundance and community composition. (A) The colonization outcome of *A. muciniphila* in different communities was persistent during serial passaging. The dashed line indicates the detection limit of the relative abundance of *A. muciniphila* (**Fig.S23**). **(B)** The community composition was stable during serial passaging. B and F denote the baseline and the final time point.

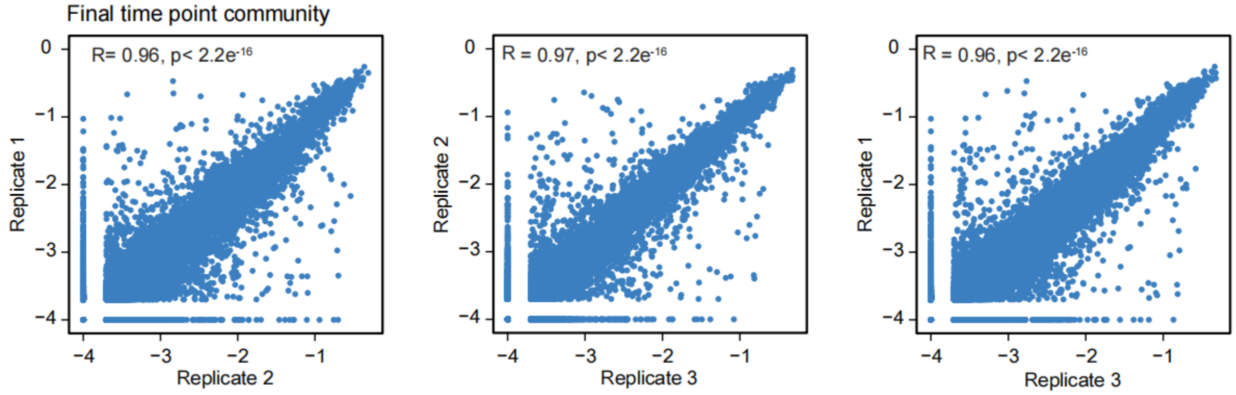

Fig. S16. The composition of *in vitro* communities post *A. muciniphila* invasion is highly reproducible across replicates. The species-level compositional profile of the post-invasion communities is highly reproducible among technical replicates (Pearson's correlation). For visualization, the relative abundance was set to 10^{-4} if it was below the detection limit. n= 3 replicates.

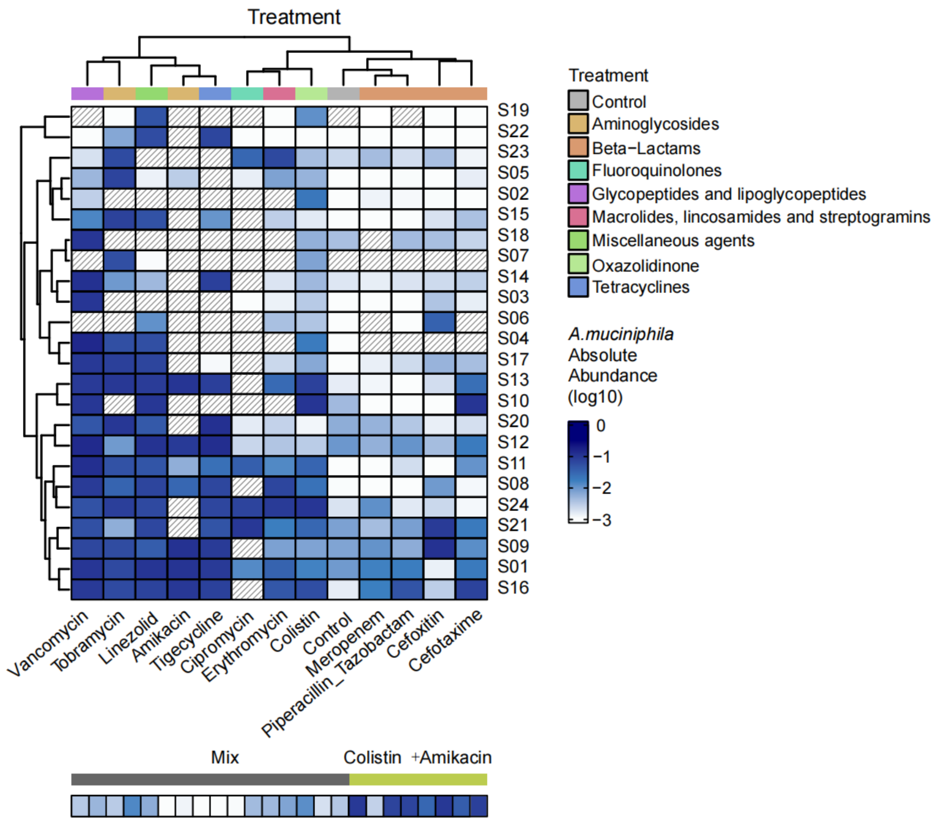

Fig. S17. Variations in the colonization outcomes of *A. muciniphila* across different donors and antibiotics treatments. For instance, post-invasion abundance of *A. muciniphila* in communities derived from donor S16 was higher than other donors; post-invasion abundance of *A. muciniphila* in communities treated with vancomycin was higher than the control group and other treatment groups. Each row corresponds to a donor from which the communities were derived, each column corresponds to a treatment. The color gradient represents absolute abundance (OD_{600}×relative abundance) of *A. muciniphila* at the post-invasion steady state. Samples marked with slashes were not used in *A. muciniphila* invasion experiments. Mix indicates the group of communities derived from mixing two different donors.

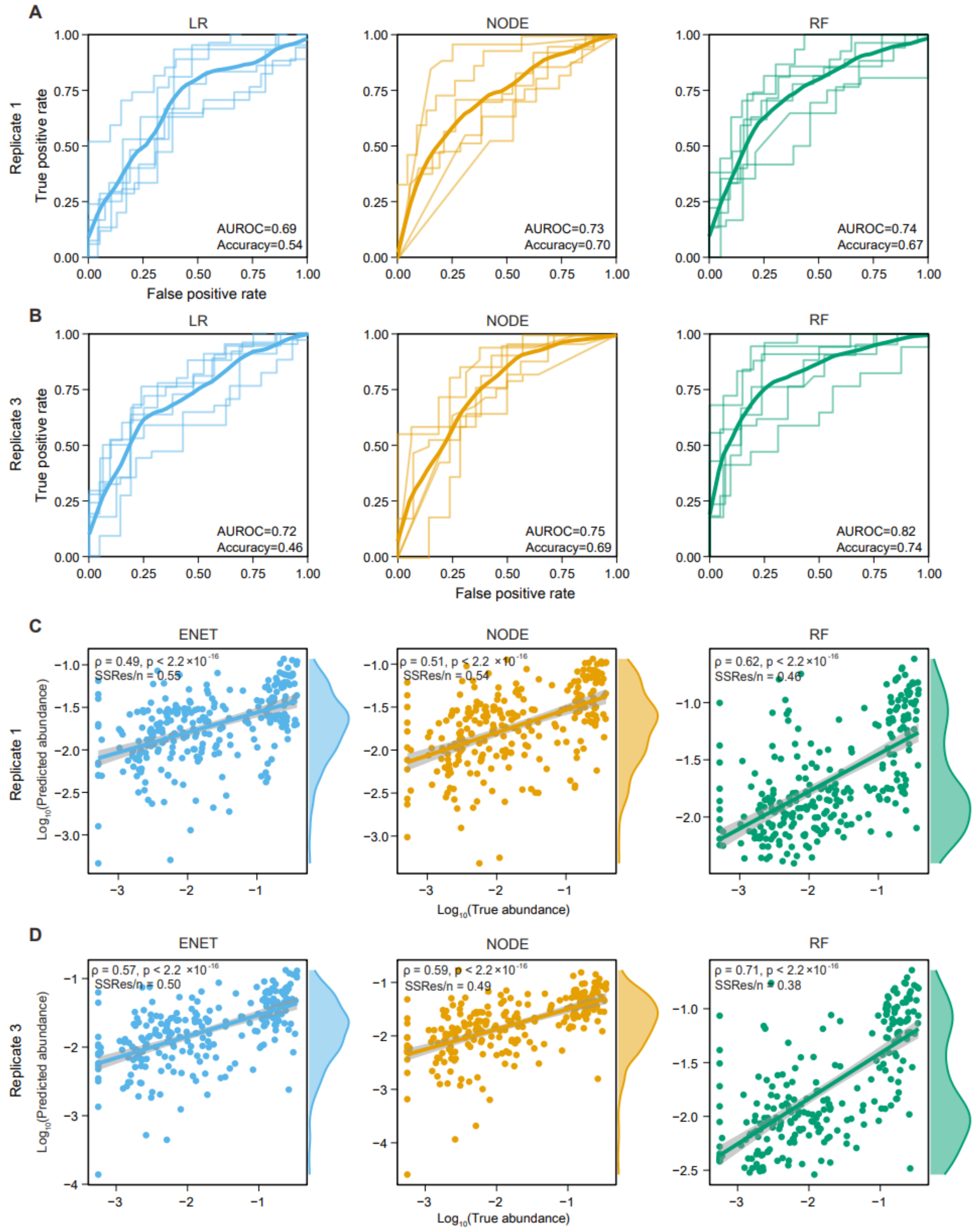

Fig. S18. The performance of colonization outcome prediction for *A. muciniphila* is consistent across replicates. ROC curve of machine learning models in binary classification (high permissive vs. Low permissive) of the colonization outcomes of *A. muciniphila* in replicate 1 **(A)** and replicate

3 **(B)**. For each 6-fold cross validation (ROC curves shown in light color), we trained each model using the samples from 20 subjects and the samples from the remaining 4 subjects to evaluate the model. The mean ROC curve is shown in dark color. Pearson's correlation coefficient and the average squared differences between the predicted and the observed abundance (log-transformed values) of *A. muciniphila* in replicate 1 **(C)** and replicate 3 **(D)**. LR: Logistic Regression, ENET: Elastic Net Linear Regression, NODE: COP-Neural Ordinary Differential Equations regressor, RF: Random Forest regressor.

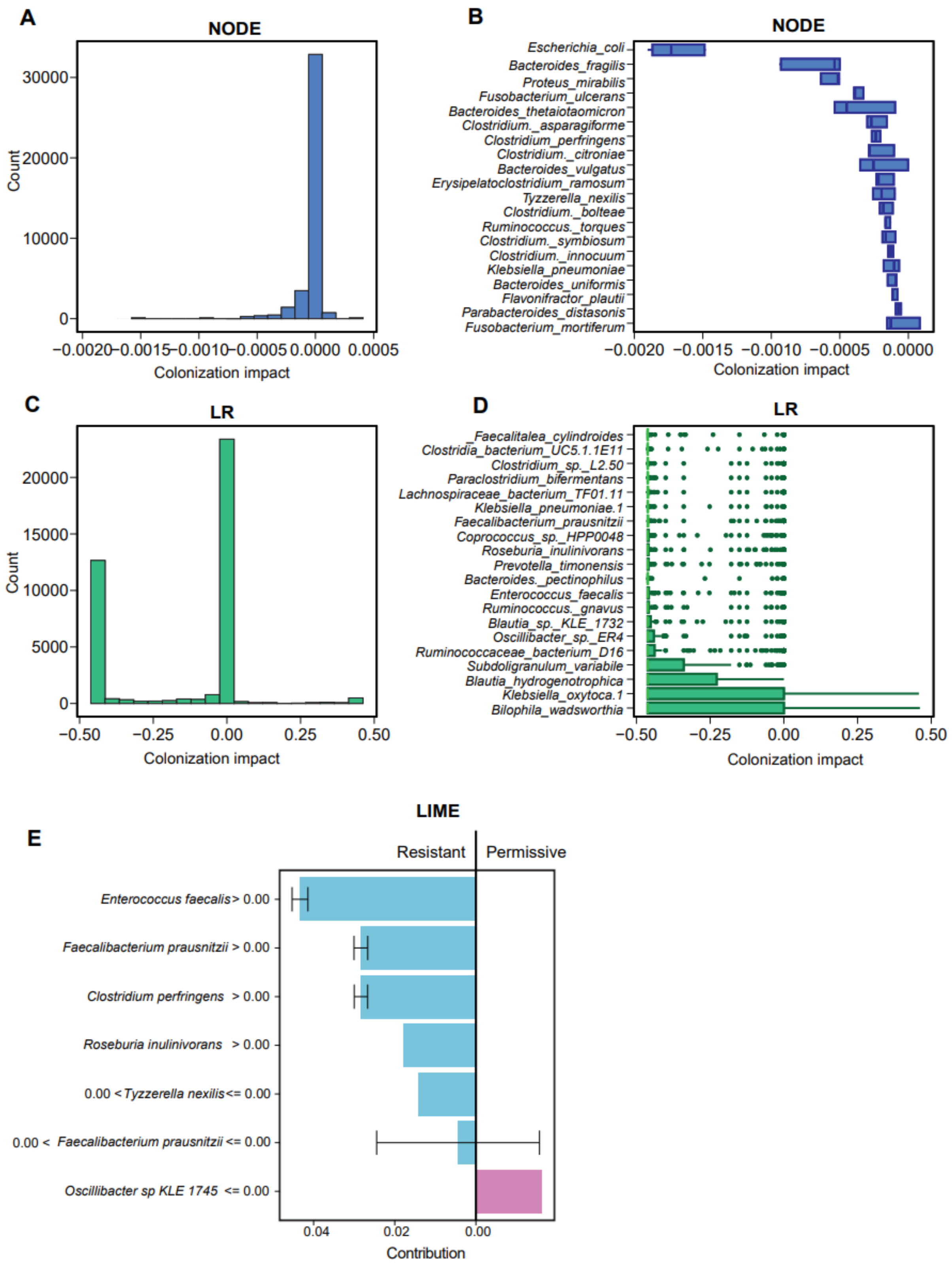

Fig. S19. Colonization impact in experimental communities predicted by NODE and logistic regression. (A-B) The distribution of colonization impact on *E. faecium*, and the top-ranking species with negative colonization impact (median across different communities) predicted by

NODE regression. **(C-D)** The distribution of colonization impact on *E. faecium*, and the top-ranking species with negative colonization impact (median across different communities) predicted by logistic regression. **(E)** The top features of the Random Forest classifier identified by LIME. We use LIME to interpret the random forest prediction for *E. faecium* colonization, with 80% as the training set and the remaining 20% as the test set. For each test sample, we selected the top 5 features and showed the mean and standard deviation of each feature among all test samples.

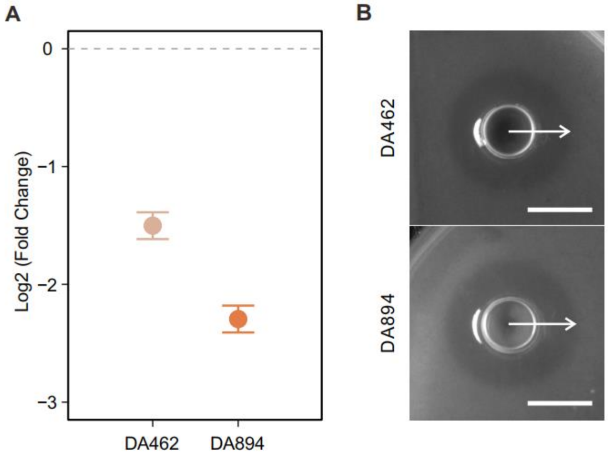

Fig. S20. *E. faecalis* inhibits the growth of *E. faecium* in pairwise co-culture. (A) The fold change in the abundance of *E. faecium* (the pairwise co-culture group divided by the mono-culture group) was lower than 1 (dashed line), indicating that the growth of *E. faecium* was inhibited in the presence of *E. faecalis* during pairwise co-culture in BHI. n= 3 replicates, the error bars are SEMs, measured by qPCR. **(B)** The Oxford cup assay was used to determine the inhibition of *E. faecium* by *E. faecalis.* An inhibition zone surrounding the Oxford Cup when *E. faecalis* was present. Scale bar, 1 cm. Two *E. faecalis* strains DA462 and DA894 were used in the assays.

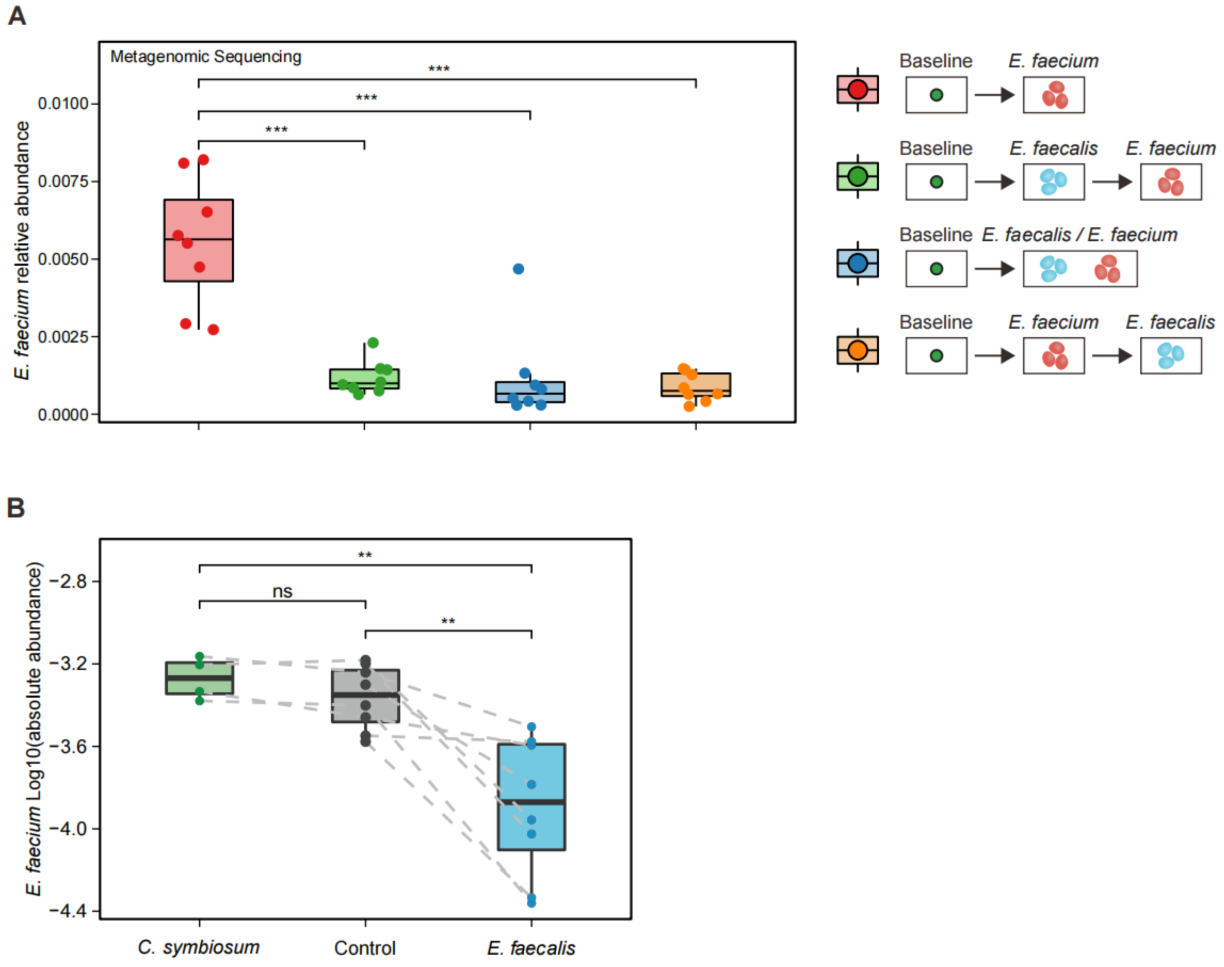

Fig. S21. *E. faecalis* inhibits the growth of *E. faecium* in human stool-derived *in vitro* communities. (A) The end-point abundance of *E. faecium*, measured by metagenomic sequencing. **(B)** The end-point abundance of *E. faecium* in communities inoculated with *E. faecalis* (inhibitory) or *C. symbiosum* (neutral) before *E. faecium* invasion (ns, not significant, ** $p < 0.01$, *** $p < 0.001$, Mann-Whitney U-tests).

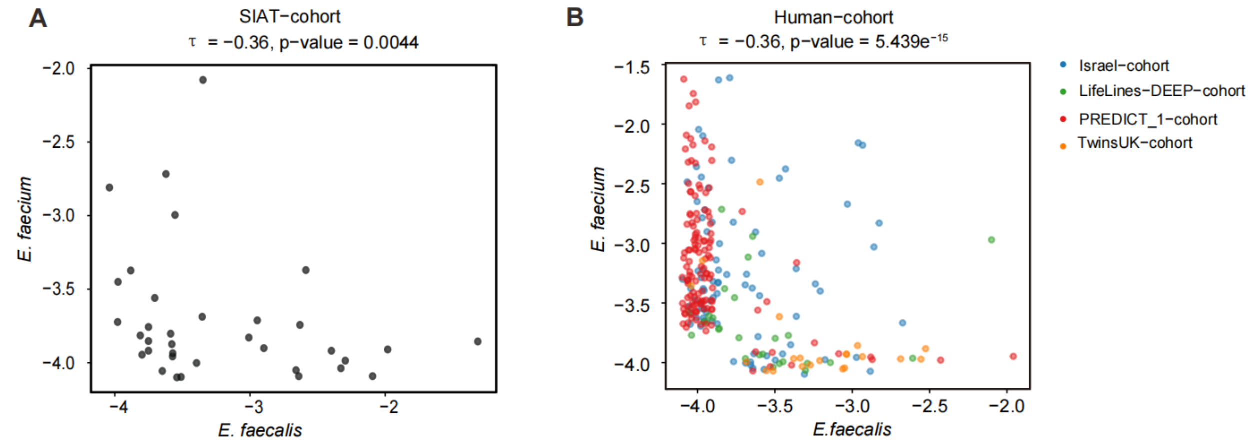

Fig. S22. The relative abundance of *E. faecalis* and *E. faecium* is negatively correlated in human gut metagenomic samples. (A) Negative correlation (Kendall correlation τ = -0.36) between the relative abundances of *E. faecalis* and *E. faecium* in the SIAT cohort. **(B)** Negative correlation (Kendall correlation τ = -0.36) between the relative abundances of *E. faecalis* and *E. faecium* in independent human cohorts. The detection limit in relative abundance was set to 10^{-4}. 71.5% of the samples in the SIAT cohort and 93.8% of the samples in the four independent cohorts were negative (i.e., below the detection limit) for both *E. faecium* and *E. faecalis.*

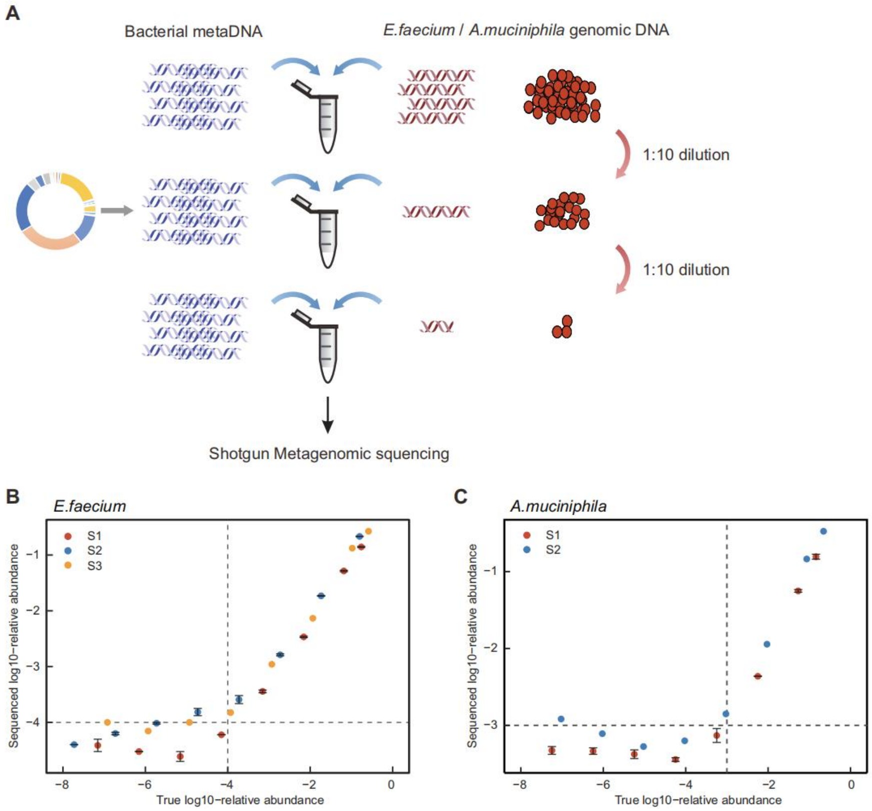

Fig. S23. Quantification of the relative abundance of *E.faecium* and *A.muciniphila* by metagenomic sequencing. (A) To confirm the accuracy of shallow metagenomic sequencing in quantifying the relative abundance of *E. faecium* and *A. muciniphila*, a spike-in experiment was conducted. The spike-in DNA of the target species (*E. faecium* or *A. muciniphila*) was 1:10 diluted for eight times and was added to the microbial metaDNA to a mixed DNA sample. The mixed DNA was then used for library construction and metagenomic sequencing. **(B-C)** By comparing the detected relative abundance generated by shallow metagenomic sequencing with the expected abundance, the accuracy and sensitivity of our workflow were determined. The detection threshold of *E. faecium* is 0.0001 and the detection threshold of *A. muciniphila* is 0.001.

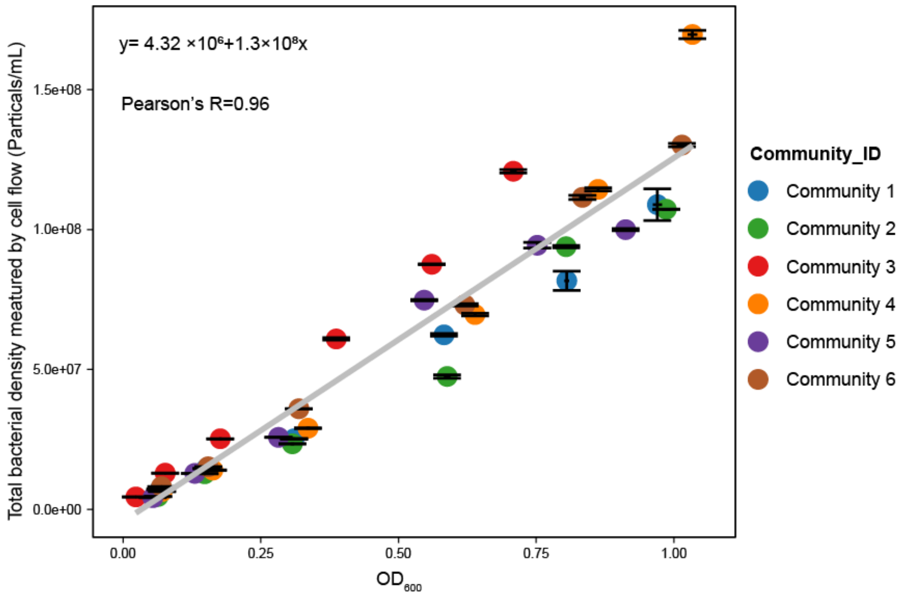

Fig. S24. Optical density measurements (OD_{600}) is highly correlated to cell densities derived by flow cytometry. Data were collected using six different stool-derived communities. The error bars indicate the standard error for each community (n=3 replicates).

Supplementary Table S1

Information of antibiotics used in this study.

Antibiotics	Name	Concentration（ug/ml）	Target	Class
Abx1	Meropenem	35	Cell wall	beta-lactams
Abx2	Cefoxitin	10	Cell wall	beta-lactams
Abx3	Cefotaxime sodium salt	40	Cell wall	beta-lactams
Abx4	Piperacillin Sodium+Tazobactam acid	20+2.5	Cell wall	beta-lactams
Abx5	Vancomycin	16	Cell wall	Glycopeptides and lipoglycopeptides
Abx6	Colistin sulfate salt	20	Cell wall	Miscellaneous agents
Abx7	Ciprofloxacin	12	DNA synthesis	(Fluoro-)quinolones
Abx8	Tobramycin	400	Protein synthesis	Aminoglycosides
Abx9	Amikacin	200	Protein synthesis	Aminoglycosides
Abx10	Erythromycin	80	Protein synthesis	Macrolides, lincosamides and streptogramins
Abx11	Linezolid	70	Protein synthesis	Oxazolidinone
Abx12	Tigecycline	0.256	Protein synthesis	Tetracyclines

Supplementary References

1. An Illustrated Guide to Theoretical Ecology. *Journal of Mammalogy* **82**, 247-248 (2001).
2. Erdős, P., Alfréd Rényi On the evolution of random graphs. *Publ. Math. Inst. Hung. Acad. Sci* **5**, 43 (1960).
3. Michel-Mata, S., Wang, X.W., Liu, Y.Y. & Angulo, M.T. Predicting microbiome compositions from species assemblages through deep learning. *iMeta* **1** (2022).
4. Paszke, A.a.G., Sam and Massa, Francisco and Lerer, Adam and Bradbury, James and Chanan, Gregory and Killeen, Trevor and Lin, Zeming and Gimelshein, Natalia and Antiga, Luca and Desmaison, Alban and Kopf, Andreas and Yang, Edward and DeVito, Zachary and Raison, Martin and Tejani, Alykhan and Chilamkurthy, Sasank and Steiner, Benoit and Fang, Lu and Bai, Junjie and Chintala, Soumith PyTorch: An Imperative Style, High-Performance Deep Learning Library. *33rd Conference on Neural Information Processing Systems (NeurIPS 2019)* (2019).
5. Buitinck L, L.G., Blondel M, Pedregosa F, Mueller A, Grisel O, Niculae V, Prettenhofer P, Gramfort A, Grobler J, Layton R API design for machine learning software: experiences from the scikit-learn project. *arXiv preprint* **arXiv:1309.0238** (2013).
6. Dehbashi, S., Tahmasebi, H., Sedighi, P., Davarian, F. & Arabestani, M.R. Development of high-resolution melting curve analysis in rapid detection of vanA gene, Enterococcus faecalis, and Enterococcus faecium from clinical isolates. *Trop Med Health* **48**, 8 (2020).
7. Zeevi, D. et al. Personalized Nutrition by Prediction of Glycemic Responses. *Cell* **163**, 1079-1094 (2015).
8. Bonder, M.J. et al. The effect of host genetics on the gut microbiome. *Nature genetics* **48**, 1407-1412 (2016).
9. Asnicar, F. et al. Microbiome connections with host metabolism and habitual diet from 1,098 deeply phenotyped individuals. *Nat Med* **27**, 321-332 (2021).
10. Xie, H. et al. Shotgun Metagenomics of 250 Adult Twins Reveals Genetic and Environmental Impacts on the Gut Microbiome. *Cell Syst.* **3**, 572-584 e573 (2016).
11. Gibson, T.E., Bashan, A., Cao, H.T., Weiss, S.T. & Liu, Y.Y. On the Origins and Control of Community Types in the Human Microbiome. *PLoS Comput Biol* **12**, e1004688 (2016).
12. Bunin, G. in Physical Review E, Vol. 95 1-8 (2017).
