# Supplementary material for: Data-driven prediction of colonization outcomes for complex microbial communities
Source: Nat Commun. 2024 Mar 16;15:2406. doi: 10.1038/s41467-024-46766-y (PMC10944475; doi:10.1038/s41467-024-46766-y)
Supplement: Supplementary file 2 — Reporting Summary [file 41467_2024_46766_MOESM2_ESM.pdf]

nature portfolio

Corresponding author(s): Yang-Yu Liu, Lei Dai

Last updated by author(s): Feb 28, 2024

Reporting Summary

Nature Portfolio wishes to improve the reproducibility of the work that we publish. This form provides structure for consistency and transparency in reporting. For further information on Nature Portfolio policies, see our Editorial Policies and the Editorial Policy Checklist.

Statistics

For all statistical analyses, confirm that the following items are present in the figure legend, table legend, main text, or Methods section.

n/a	Confirmed	
☐	☒	The exact sample size (n) for each experimental group/condition, given as a discrete number and unit of measurement
☐	☒	A statement on whether measurements were taken from distinct samples or whether the same sample was measured repeatedly
☐	☒	The statistical test(s) used AND whether they are one- or two-sided<br>*Only common tests should be described solely by name; describe more complex techniques in the Methods section.*
☒	☐	A description of all covariates tested
☐	☒	A description of any assumptions or corrections, such as tests of normality and adjustment for multiple comparisons
☐	☒	A full description of the statistical parameters including central tendency (e.g. means) or other basic estimates (e.g. regression coefficient) AND variation (e.g. standard deviation) or associated estimates of uncertainty (e.g. confidence intervals)
☐	☒	For null hypothesis testing, the test statistic (e.g. F, t, r) with confidence intervals, effect sizes, degrees of freedom and P value noted<br>*Give P values as exact values whenever suitable.*
☒	☐	For Bayesian analysis, information on the choice of priors and Markov chain Monte Carlo settings
☒	☐	For hierarchical and complex designs, identification of the appropriate level for tests and full reporting of outcomes
☐	☒	Estimates of effect sizes (e.g. Cohen's d, Pearson's r), indicating how they were calculated

Our web collection on statistics for biologists contains articles on many of the points above.

Software and code

Policy information about availability of computer code

Data collection	R pacakge "seqtime" (version 0.1.1) was used to generate simulated datasets.
Data analysis	Python (3.8.13) and Pytorch (version 1.7.1) were used to train NODE. Random Forest and Linear Regression were implemented using scikit-learn (version 1.3). Microbial community composition from metagenomic sequencing data was generated using the SHOGUN pipeline and the RefSeq database version 82; R pacakge "vegan" (version 2.6-4) was used to analysis microbiota community composition characteristics. The code for simulation and data analysis is available at https://github.com/spxuw/COP.

For manuscripts utilizing custom algorithms or software that are central to the research but not yet described in published literature, software must be made available to editors and reviewers. We strongly encourage code deposition in a community repository (e.g. GitHub). See the Nature Portfolio guidelines for submitting code & software for further information.

Data

Policy information about availability of data

All manuscripts must include a data availability statement. This statement should provide the following information, where applicable:

- Accession codes, unique identifiers, or web links for publicly available datasets
- A description of any restrictions on data availability
- For clinical datasets or third party data, please ensure that the statement adheres to our policy

All sequencing data generated in this study are available from European Nucleotide Archive(ENA) under study accession number PRJEB60398(https://www.ebi.ac.uk/ena/browser/view/PRJEB60398). Sample accession code, metadata and related source data are provided as a Source Data file with this paper.

Research involving human participants, their data, or biological material

Policy information about studies with human participants or human data. See also policy information about sex, gender (identity/presentation), and sexual orientation and race, ethnicity and racism.

Reporting on sex and gender	Samples generated in vitro were not sex or gender specific. The study included a total of 24 human donor subjects, comprising 7 females and 17 males.
Reporting on race, ethnicity, or other socially relevant groupings	The 24 healthy individuals from Shenzhen, China belong to the Han Chinese ethnic group.
Population characteristics	The study included healthy individuals between the ages of 18 and 65 who were capable of providing written informed consent. A total of 119 individuals from Shenzhen, China, known as the SIAT cohort, were recruited for the study. From the fecal samples obtained from the SIAT cohort, we selected 24 donors who were found to have no detection of E. faecium and A. muciniphila through metagenomic sequencing.
Recruitment	Exclusion criteria for the cohort included antibiotic exposure in the past 3 months for any reason,any chronic diseases known to affect the gut microbiota, i.e.,diabetes,obesity and other digestive tract disease. There were no self-selection bias that is likely to impact results.
Ethics oversight	The collection of human stool samples from volunteers at SIAT (referred to as “SIAT cohort”) were approved by the Shenzhen Institute of Advanced Technology, Chinese Academy of Sciences (SIAT-IRB-200315-HO438).

Note that full information on the approval of the study protocol must also be provided in the manuscript.

Field-specific reporting

Please select the one below that is the best fit for your research. If you are not sure, read the appropriate sections before making your selection.

[x] Life sciences [ ] Behavioural & social sciences [ ] Ecological, evolutionary & environmental sciences

For a reference copy of the document with all sections, see nature.com/documents/nr-reporting-summary-flat.pdf

Life sciences study design

All studies must disclose on these points even when the disclosure is negative.

Sample size	Sample size was determined by the requirement of the machine learning model, e.g., the number of the samples is approximately twice of the number of features. We addressed this sample size question in the results section(Fig 1).
Data exclusions	No samples were excluded.
Replication	We have compared the community composition and the prediction performance to demonstrate that the findings are consistent across three replicates.
Randomization	Randomization was not relevant for this study. The samples do not have group assignments. We applied the cross-validation technique to ensure that all samples were included in training data and testing data for the machine learning models.
Blinding	Blinding was not applied in this study. The experiments were carried out in vitro and do not involve human subjects.

Reporting for specific materials, systems and methods

We require information from authors about some types of materials, experimental systems and methods used in many studies. Here, indicate whether each material, system or method listed is relevant to your study. If you are not sure if a list item applies to your research, read the appropriate section before selecting a response.

Materials & experimental systems

n/a	Involved in the study
[x]	[ ] Antibodies
[x]	[ ] Eukaryotic cell lines
[x]	[ ] Palaeontology and archaeology
[x]	[ ] Animals and other organisms
[x]	[ ] Clinical data
[x]	[ ] Dual use research of concern
[x]	[ ] Plants

Methods

n/a	Involved in the study
[x]	[ ] ChIP-seq
[x]	[ ] Flow cytometry
[x]	[ ] MRI-based neuroimaging
