# Supplementary material for: Data-driven prediction of colonization outcomes for complex microbial communities
Source: Nat Commun. 2024 Mar 16;15:2406. doi: 10.1038/s41467-024-46766-y (PMC10944475; doi:10.1038/s41467-024-46766-y)
Supplement: Supplementary file 5 — Description of Additional Supplementary Files [file 41467_2024_46766_MOESM5_ESM.pdf]

Description of Additional Supplementary Files

Supplementary Data 1. Raw data availability in this study.

Supplementary Data 2. Metadata for the sequenced samples in this study.
